# Supplementary material for: Urinary Metabolome Analyses of Patients with Acute Kidney Injury Using Capillary Electrophoresis-Mass Spectrometry
Source: Metabolites. 2021 Sep 30;11(10):671. doi: 10.3390/metabo11100671 (PMC8540909; doi:10.3390/metabo11100671)
Supplement: Supplementary file 1 [file metabolites-11-00671-s001.zip › metabolites-1390119-supplementary.pdf]

(Figure S1)

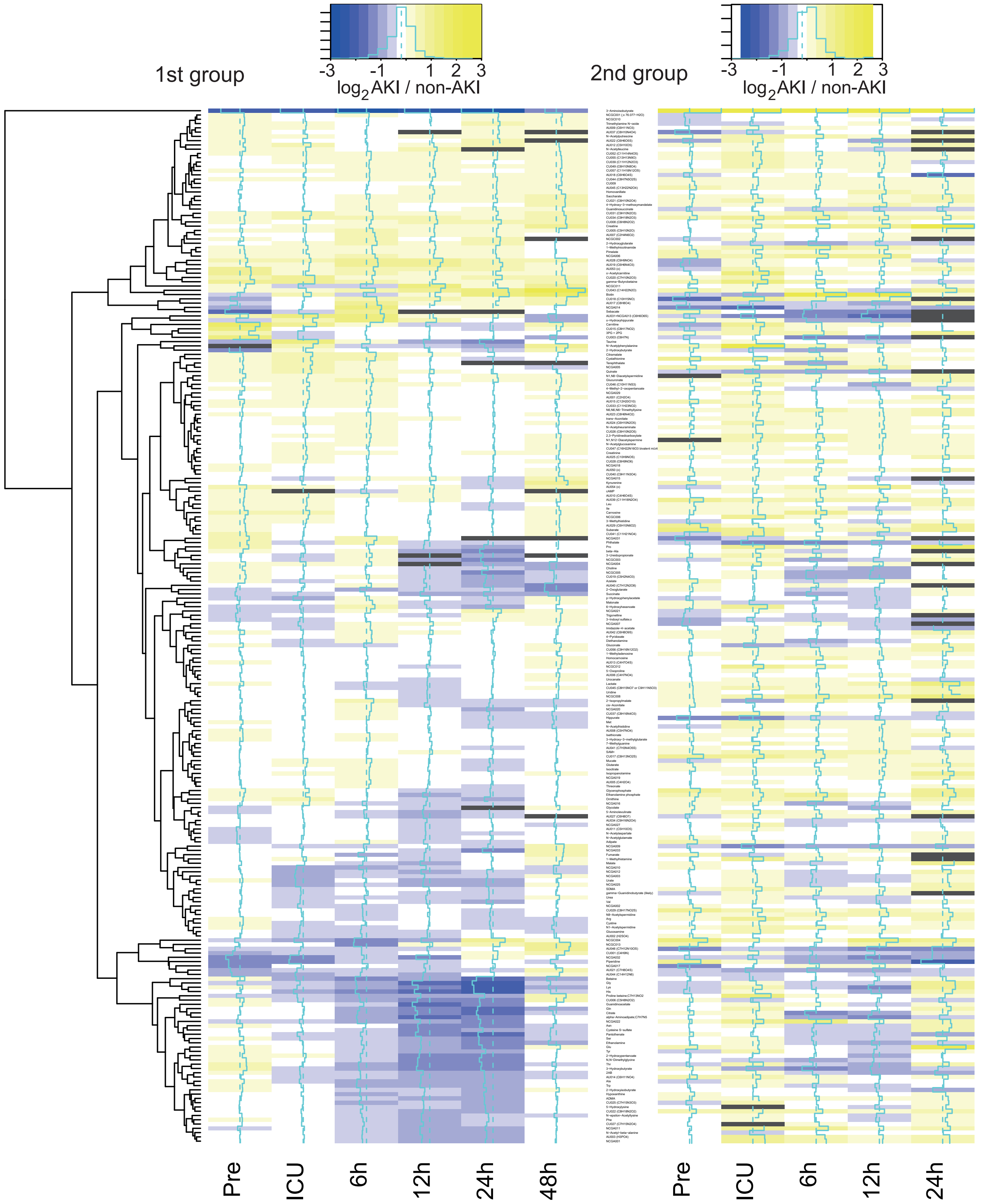

Figure S1: Log2-ratio of metabolome profiles for AKI vs non-AKI

Results for both known and uncharacterized metabolites are shown. For the results that focus only on known metabolites, see Figure 1b.

(Figure S2)

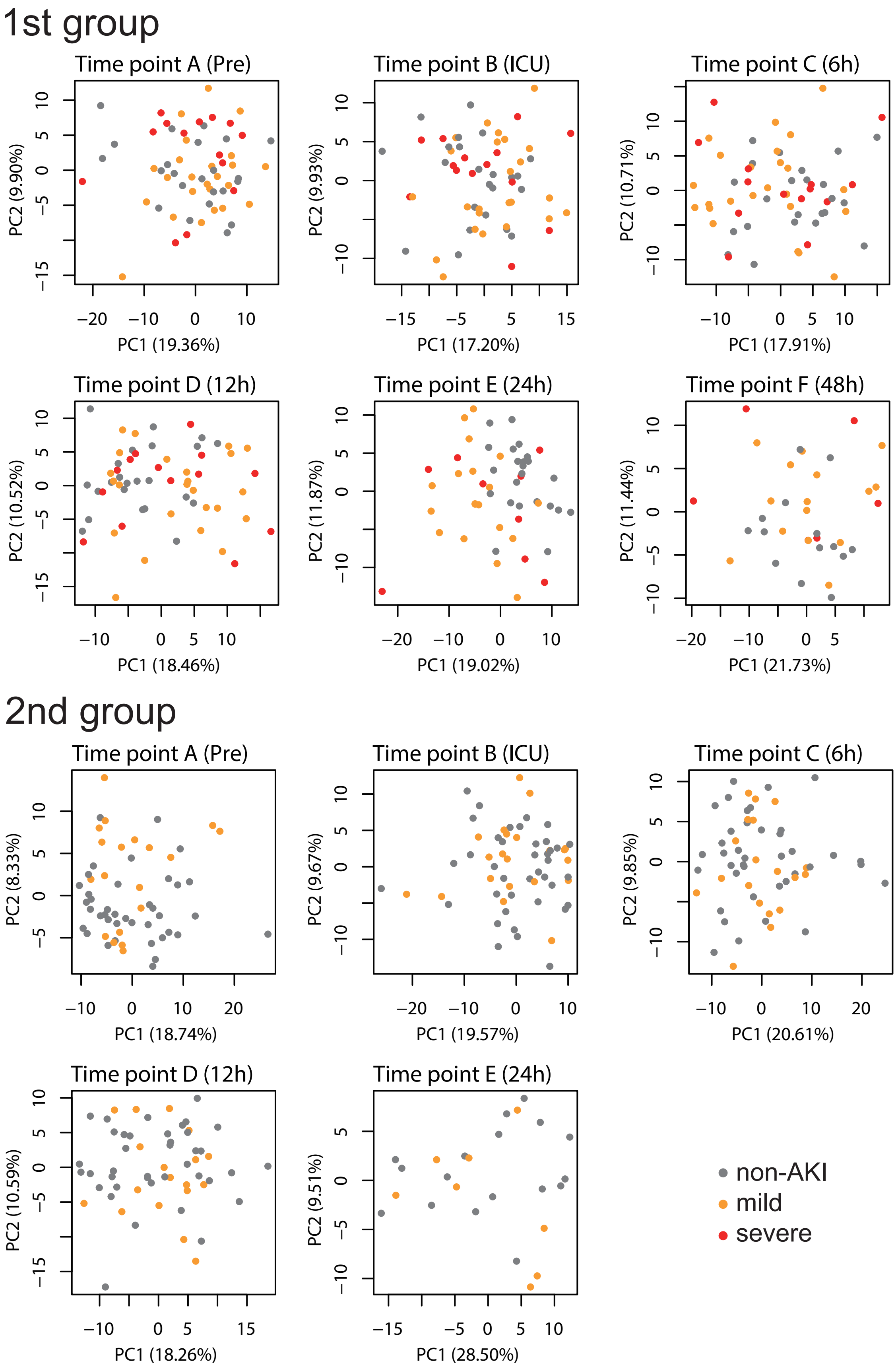

Figure S2: Principal component analysis (PCAs) of metabolome profiles at each time point in the two subject groups

(Figure S3)

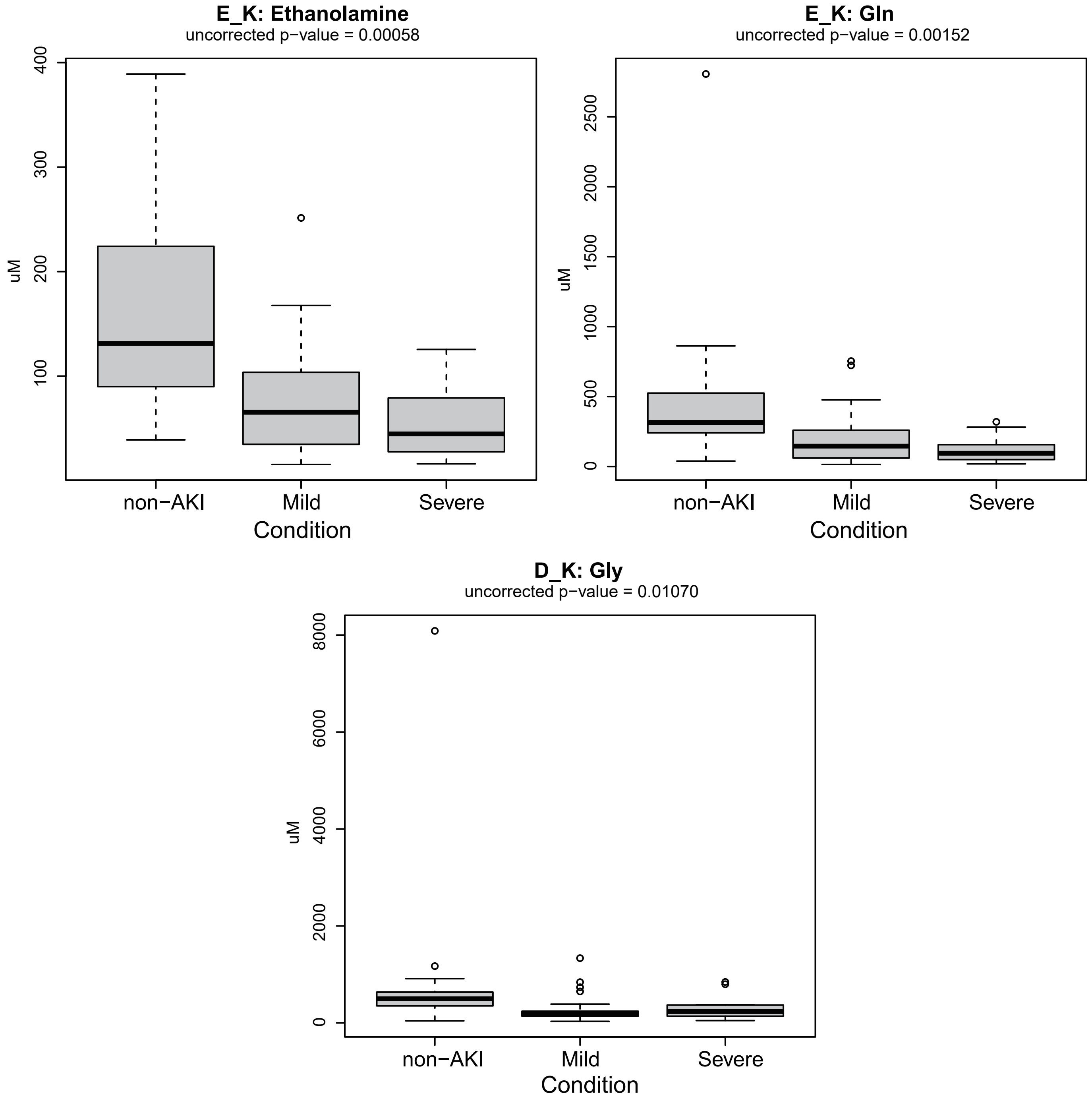

Figure S3: Non-normalized metabolite levels of ethanolamine and glutamine at 24 h and glycine at 12 h.

(Figure S4)

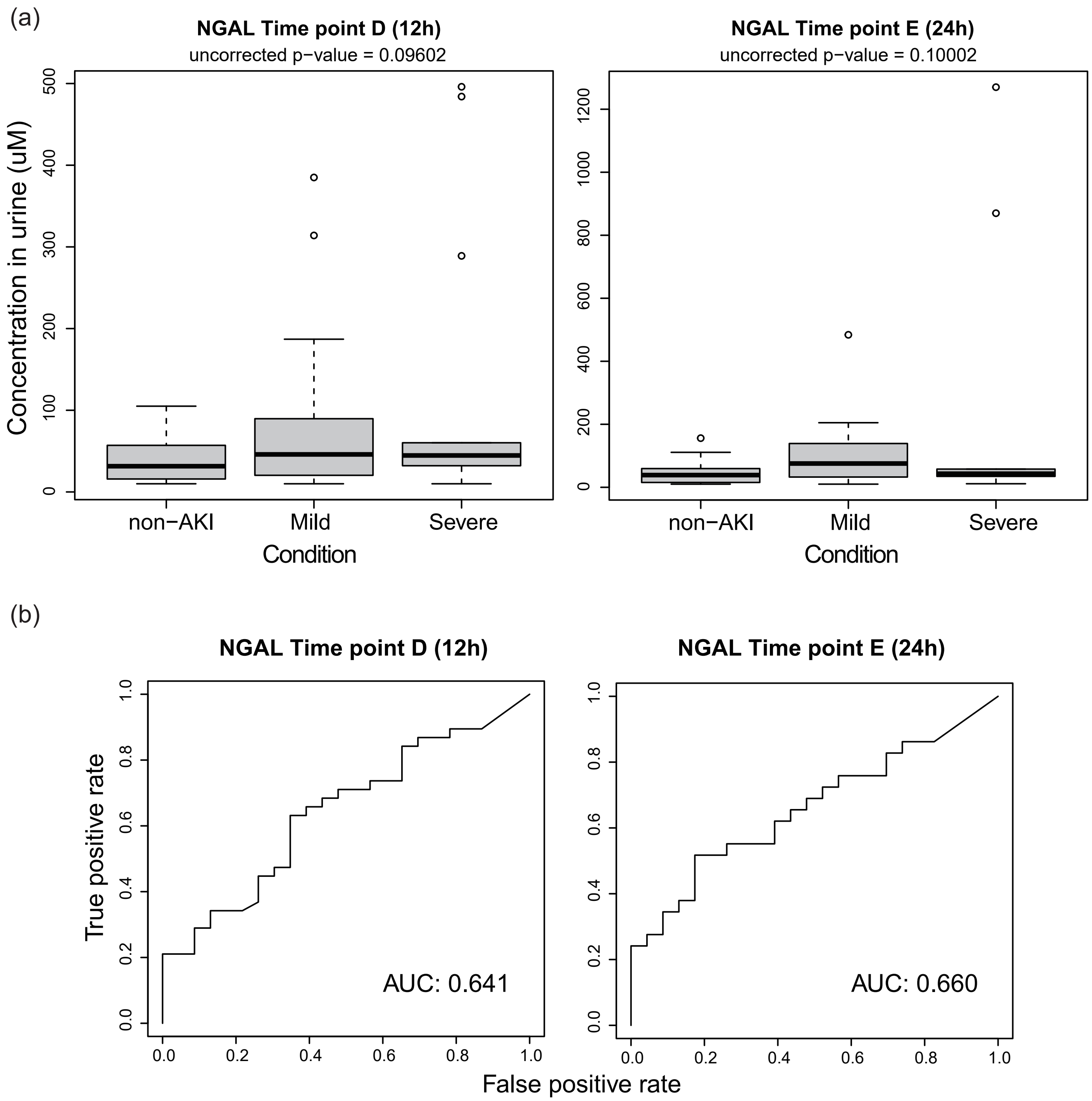

Figure S4: Quantification of urinary NGAL without urinary creatinine normalization

(a) Concentrations of urinary NGAL at 12 h (left) and 24 h (right). (b) Performance assessment of AKI prediction based on NGAL levels at 12 h (left) and 24 h (right) using ROC curves.

(Figure S5)

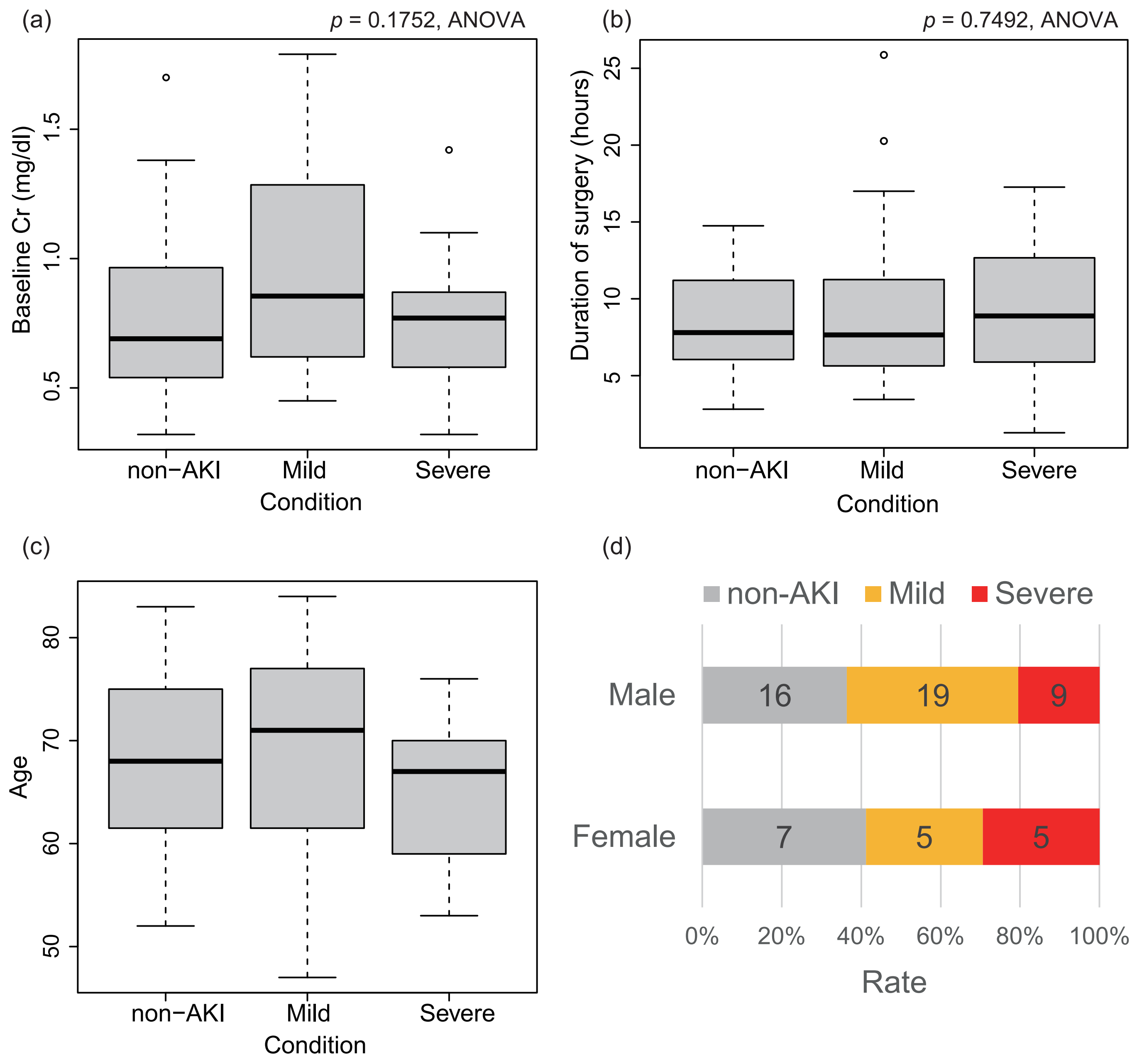

Figure S5: Distributions in clinical variables in each subject category

Distributions in baseline creatinine levels (a), duration of surgery (b) and ages (c) within each category of subjects from the 1st group. (d) Proportions of male and female subjects with non-AKI, mild and severe AKI from the 1st group. The numbers on the bars represent the numbers of subjects. Subjects for metabolome analysis were selected considering the balances in age (c) and gender (d). Other clinical variables (a and b) were not considered for selecting the subjects.

(Table S1)

Urinary Cr (mg/dL)	Dilution rate	Urine (uL)	IS + Milli-Q water (uL)	Milli-Q water (uL)	Total (uL)
100 - 300	20	20	40	340	400
50 - 100	10	20	20	160	200
25 - 50	5	20	10	70	100
10 - 25	2	20	4	16	40
0 - 10	1	90	10	0	100

Table S1: Sample dilution rate based on urinary Cr
